# HIV-1 Transcription and Latency in the Spotlight

**DOI:** 10.3390/v16020248

**Published:** 2024-02-04

**Authors:** Iván D’Orso

**Affiliations:** Department of Microbiology, The University of Texas Southwestern Medical Center, Dallas, TX 75390, USA; ivan.dorso@utsouthwestern.edu

At every integrated HIV-1 genome, there is a transcriptional cycle that ultimately shapes proviral fate [1,2,3,4]. This transcriptional cycle is regulated by viral and host factors that operate at multiple levels, from transcription initiation to elongation and termination. Our understanding of this process and its implications for latency control have evolved rapidly in the last decade. Thus, we felt the time was right to have an updated overview of the field, with a focus on recent discoveries and future research ideas. The need for these updates is further justified by the increasing recognition that both viral and host factors could be valid targets of pharmacological interventions for either permanent silencing or reactivation from latency for the elimination of reservoir cells [5].

Within this framework, in this Special Issue of *Viruses*, entitled “Regulation of HIV-1 Transcription and Latency”, we aimed to publish a series of reviews and original research articles focused on the basic mechanisms of HIV-1 transcriptional regulation; their implications for our understanding of latency establishment, maintenance, and reactivation; and applications for HIV-1 cure. Our main goal was to discuss established dogmas and provide an account of the recent discoveries in this rapidly growing field. This issue contains three research articles focused on factors that control HIV-1 latency reactivation and seven review articles focused on cure approaches, HIV-1 silencing, and reactivation.

Four reviews discuss the nuances of the various ongoing HIV-1 eradication approaches. First, Browne, Margolis, and colleagues describe the differences between latency reversal and latency prevention for the therapeutic manipulation of HIV-1 transcription and latency. While the initial efforts toward a functional cure have relied on latency reversal, most recent work has demonstrated that the majority of the long-lived latent reservoir is established early during ART initiation, thus sharing the encouraging news about the therapeutic value of early targeting the reservoir. By reducing the formation of a sizable fraction of the latent reservoir with ART initiation, subsequent treatment with latency-reversing agents alongside immune clearance agents may be more effective as dual prevention and elimination treatments. Then, Pache, Chanda, and colleagues discuss “Breaking the Silence” as their view toward a road to a cure. Besides the known block-and-lock and shock-and-kill approaches, they provide a summary of the most current promising cure avenues, including both latency-promoting agents (LPAs) and latency-reversing agents (LRAs), that have shown promise ex vivo and in vivo. In another contribution, Ott, Valente, and colleagues propose a novel “block–lock–stop” approach involving durable silencing of viral transcription toward an irreversible transcriptionally inactive state to achieve long-term ART-free control of HIV-1. A transformational acceleration of transcriptionally competent to incompetent proviruses is proposed, imitating the path of endogenous retrovirus silencing during millions of years of evolution. Finally, Howard and Bosque provide a thorough description of the gamma cytokine IL-15 and its super agonist N-803, which are currently under clinical investigation. These have been shown to mutually reactivate latent HIV-1 and enhance immune effector functions, which are both required for the reduction in the latent reservoir. These are then followed by a review by Kearney and colleagues, who discuss the select genetic, epigenetic, cellular, and immunological determinants of viral transcriptional suppression and the clonal expansion of HIV-1 in reservoir CD4^+^ T cell clones, plus the interdependencies among these features and implications for HIV-1 persistence.

Two studies then touch upon the use of mathematical modeling for understanding the regulation of HIV-1 transcription. First, Basyuk, Bertrand, and colleagues elaborate on the importance of transcriptional stochasticity, due to alternate on and off states of the viral promoter, as a key regulatory aspect of HIV-1 transcription with a direct impact on proviral fate. In the second study, D’Orso and Forst describe the use of mathematical models to understand the dynamics of HIV-1 during productive and latent infection with emphasis on the molecular features regulating viral transcription.

As regards the research articles, Horvath and Sadowski open the discussion with a description of how upstream stimulatory factors (USFs) mutually regulate CD4^+^ T cell activation and HIV-1 latency. Their studies reveal that the genetic depletion of USF2, but not USF1, inhibits HIV-1 gene transcription in CD4^+^ T cells, while both paralogs are required for full T cell activation, unveiling unanticipated roles for this family of proteins. Then, Emerman and colleagues elegantly present an extended application of the CRISPR-HIV approach [6] to reveal that *CCNT1* encoding for the Cyclin T1 regulatory subunit of the P-TEFb kinase, crucial for transcription elongation [7], is not essential in CD4^+^ T cells but required for HIV-1 reactivation from latency, perhaps indicating a remarkable dependence of HIV-1, unlike T cell inducible genes, on Cyclin T1/P-TEFb activity. Finally, given the previous discrepancy in the field proposing that KAP1/TRIM28 is an activator of transcription required for latency reactivation and a repressor required for latency maintenance, D’Orso and colleagues revisit this discrepancy using a chemical genetics approach [8] to acutely deplete KAP1 expression to avoid the accumulation of indirect effects that can obscure the real phenotypes [9]. After validating that KAP1 is an activator and not a repressor of HIV-1 transcription, they conduct extensive deletion and mutagenesis analysis to define important protein features, including an E3 ubiquitin ligase function.

We are grateful to all authors for their expert contributions and hope that the readers of *Viruses* will treasure this issue focused on the regulation of HIV-1 transcription and latency. In closing, despite the critical importance of the HIV-1 transcriptional cycle, our understanding of the underlying mechanisms regulating proviral fate continues to be limited. This lack of complete knowledge hampers our ability to manipulate HIV-1 transcription for translational applications. We hope that funding agencies and the scientific community will still value the power of basic science research to inform on the right steps for therapeutic translations to clear the latent reservoir.
